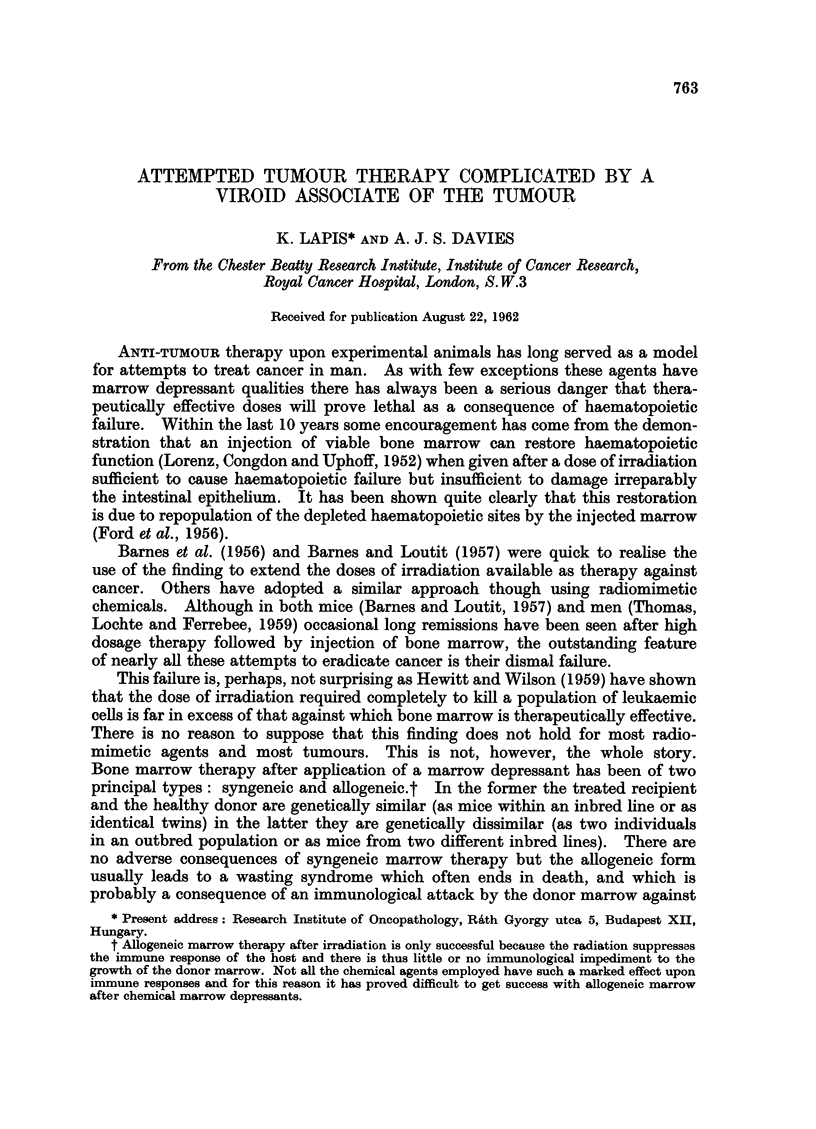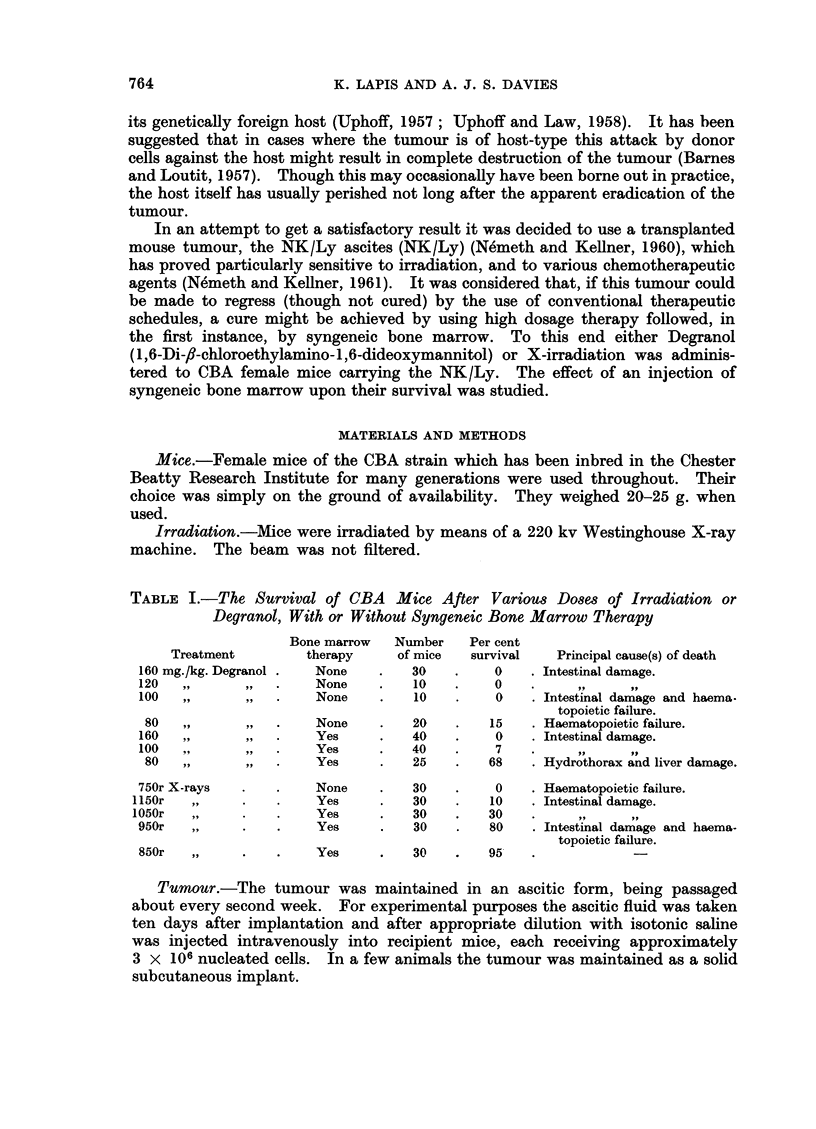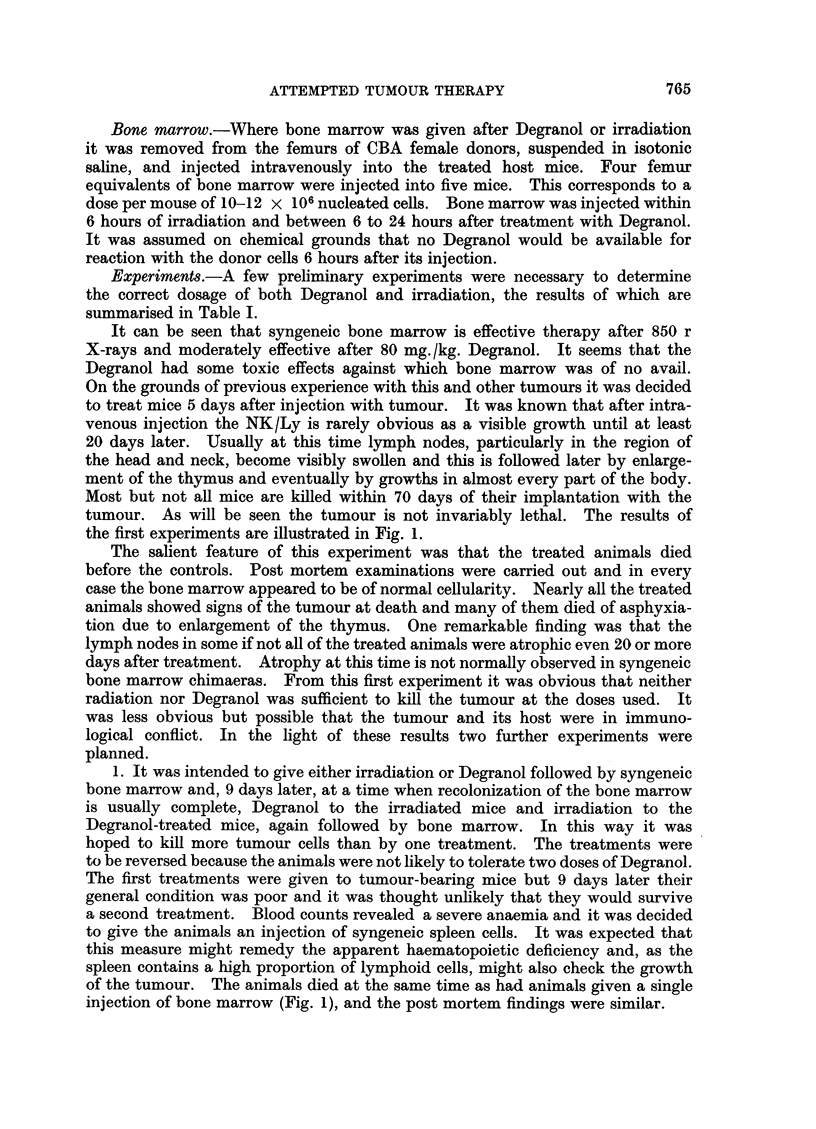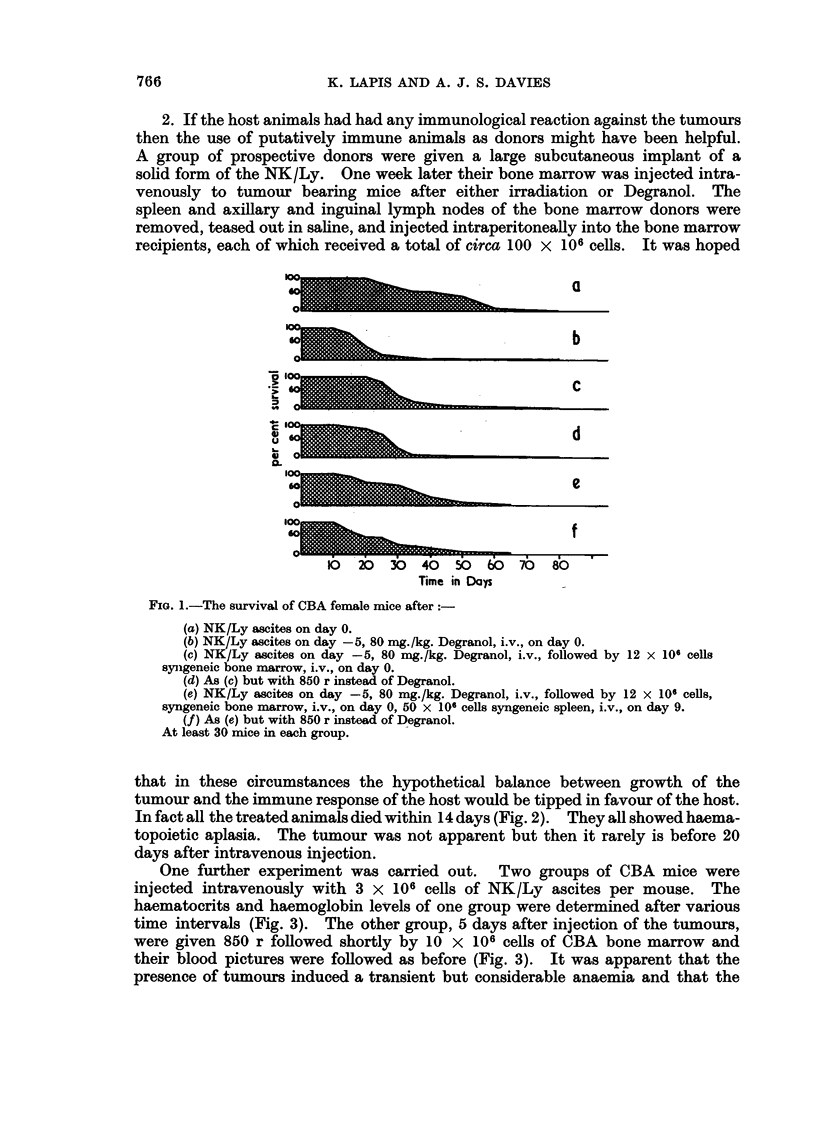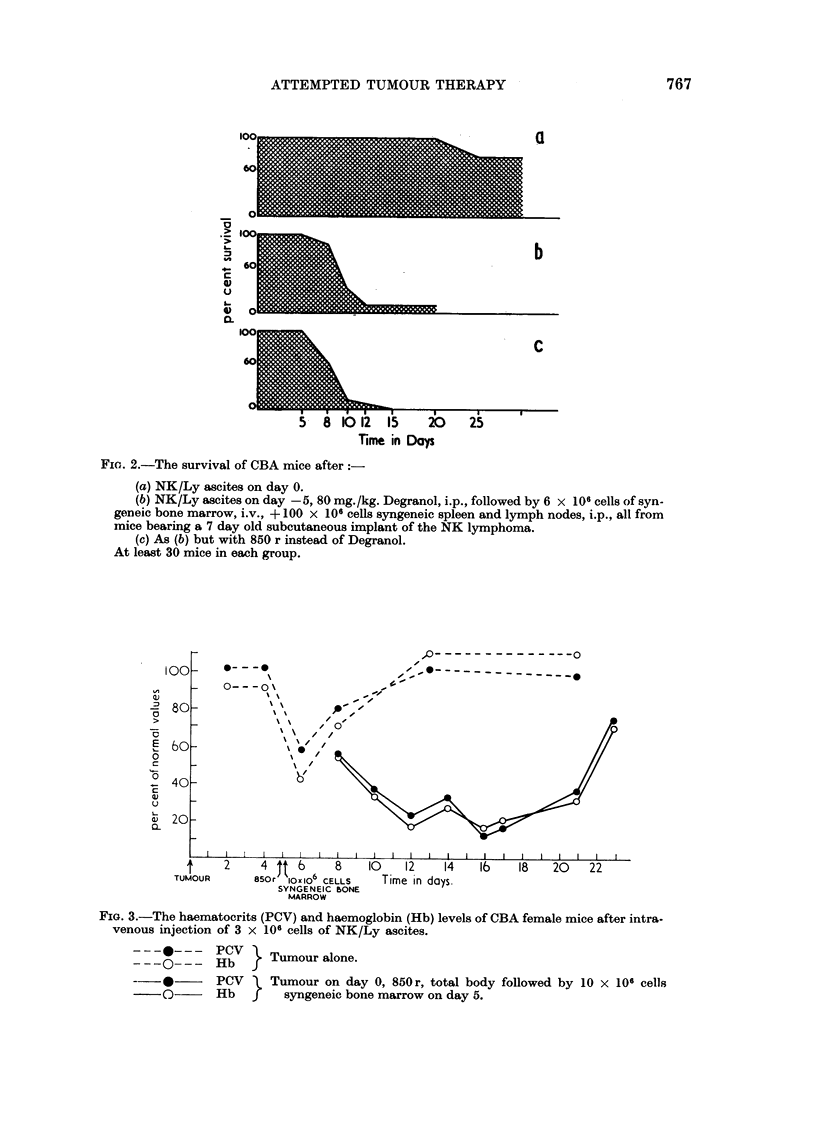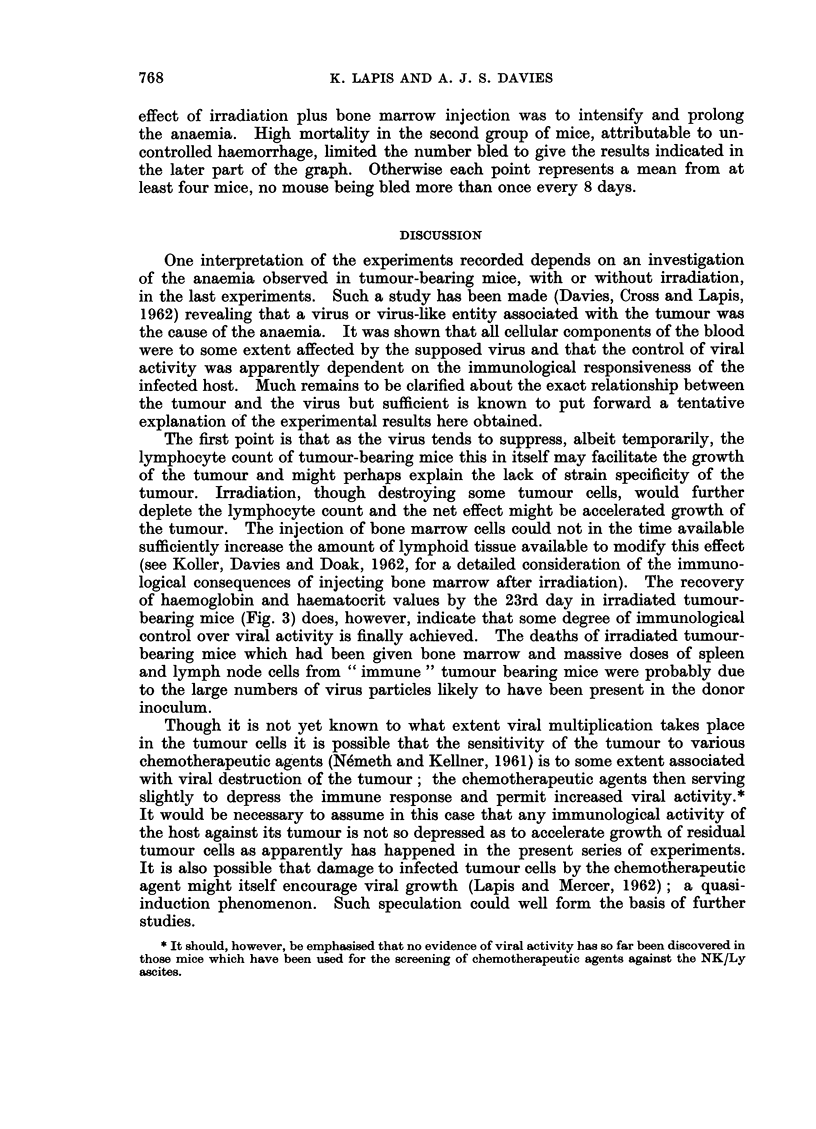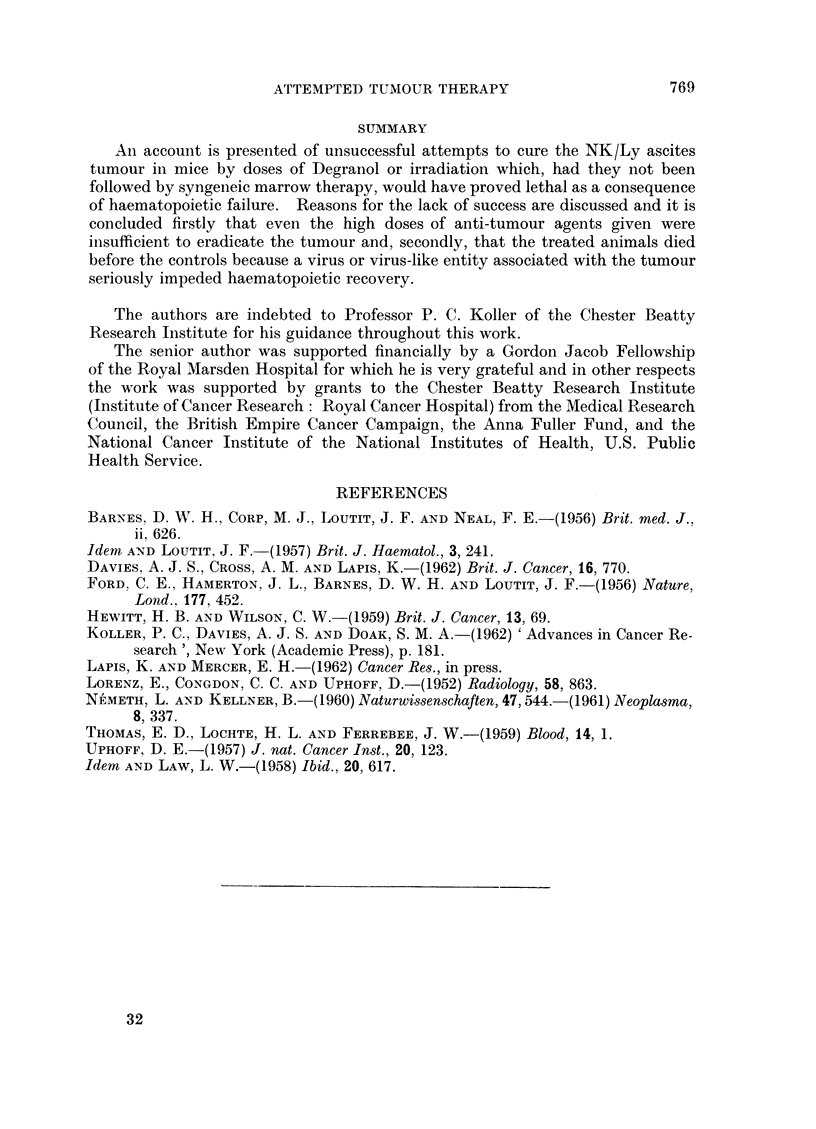# Attempted Tumour Therapy Complicated by a Viroid Associate of the Tumour

**DOI:** 10.1038/bjc.1962.89

**Published:** 1962-12

**Authors:** K. Lapis, A. J. S. Davies


### ATTEMPTED TUMOUR THERAPY COMPLICATED BY A VIROID ASSOCIATE OF THE TUMOUR

K. LAPIS* AND A. J. S. DAVIES

*From the Chester Beatty Research Institute, Institute of Cancer Research, Royal Cancer Hospital, London, S.W.3*

Received for publication August 22, 1962

ANTI-TUMOUR therapy upon experimental animals has long served as a model for attempts to treat cancer in man. As with few exceptions these agents have marrow depressant qualities there has always been a serious danger that therapeutically effective doses will prove lethal as a consequence of haematopoietic failure. Within the last 10 years some encouragement has come from the demonstration that an injection of viable bone marrow can restore haematopoietic function (Lorenz, Congdon and Uphoff, 1952) when given after a dose of irradiation sufficient to cause haematopoietic failure but insufficient to damage irreparably the intestinal epithelium. It has been shown quite clearly that this restoration is due to repopulation of the depleted haematopoietic sites by the injected marrow (Ford *et al.*, 1956).

Barnes *et al.* (1956) and Barnes and Loutit (1957) were quick to realise the use of the finding to extend the doses of irradiation available as therapy against cancer. Others have adopted a similar approach though using radiomimetic chemicals. Although in both mice (Barnes and Loutit, 1957) and men (Thomas, Lochte and Ferrebee, 1959) occasional long remissions have been seen after high dosage therapy followed by injection of bone marrow, the outstanding feature of nearly all these attempts to eradicate cancer is their dismal failure.

This failure is, perhaps, not surprising as Hewitt and Wilson (1959) have shown that the dose of irradiation required completely to kill a population of leukaemic cells is far in excess of that against which bone marrow is therapeutically effective. There is no reason to suppose that this finding does not hold for most radiomimetic agents and most tumours. This is not, however, the whole story. Bone marrow therapy after application of a marrow depressant has been of two principal types: syngeneic and allogeneic.† In the former the treated recipient and the healthy donor are genetically similar (as mice within an inbred line or as identical twins) in the latter they are genetically dissimilar (as two individuals in an outbred population or as mice from two different inbred lines). There are no adverse consequences of syngeneic marrow therapy but the allogeneic form usually leads to a wasting syndrome which often ends in death, and which is probably a consequence of an immunological attack by the donor marrow against

* Present address: Research Institute of Oncopathology, Ráth Gyorgy utca 5, Budapest XII, Hungary.

† Allogeneic marrow therapy after irradiation is only successful because the radiation suppresses the immune response of the host and there is thus little or no immunological impediment to the growth of the donor marrow. Not all the chemical agents employed have such a marked effect upon immune responses and for this reason it has proved difficult to get success with allogeneic marrow after chemical marrow depressants.

its genetically foreign host (Uphoff, 1957 ; Uphoff and Law, 1958). It has been suggested that in cases where the tumour is of host-type this attack by donor cells against the host might result in complete destruction of the tumour (Barnes and Loutit, 1957). Though this may occasionally have been borne out in practice, the host itself has usually perished not long after the apparent eradication of the tumour.

In an attempt to get a satisfactory result it was decided to use a transplanted mouse tumour, the NK/Ly ascites (NK/Ly) (Németh and Kellner, 1960), which has proved particularly sensitive to irradiation, and to various chemotherapeutic agents (Németh and Kellner, 1961). It was considered that, if this tumour could be made to regress (though not cured) by the use of conventional therapeutic schedules, a cure might be achieved by using high dosage therapy followed, in the first instance, by syngeneic bone marrow. To this end either Degranol (1,6-Di-$\beta$-chloroethylamino-1,6-dideoxymannitol) or X-irradiation was administered to CBA female mice carrying the NK/Ly. The effect of an injection of syngeneic bone marrow upon their survival was studied.

### MATERIALS AND METHODS

*Mice.*—Female mice of the CBA strain which has been inbred in the Chester Beatty Research Institute for many generations were used throughout. Their choice was simply on the ground of availability. They weighed 20–25 g. when used.

*Irradiation.*—Mice were irradiated by means of a 220 kv Westinghouse X-ray machine. The beam was not filtered.

TABLE I.—*The Survival of CBA Mice After Various Doses of Irradiation or Degranol, With or Without Syngeneic Bone Marrow Therapy*

| Treatment | Bone marrow therapy | Number of mice | Per cent survival | Principal cause(s) of death |
|---|---|---|---|---|
| 160 mg./kg. Degranol | None | 30 | 0 | Intestinal damage. |
| 120 ,, ,, | None | 10 | 0 | ,, ,, |
| 100 ,, ,, | None | 10 | 0 | Intestinal damage and haematopoietic failure. |
| 80 ,, ,, | None | 20 | 15 | Haematopoietic failure. |
| 160 ,, ,, | Yes | 40 | 0 | Intestinal damage. |
| 100 ,, ,, | Yes | 40 | 7 | ,, ,, |
| 80 ,, ,, | Yes | 25 | 68 | Hydrothorax and liver damage. |
| 750r X-rays | None | 30 | 0 | Haematopoietic failure. |
| 1150r ,, | Yes | 30 | 10 | Intestinal damage. |
| 1050r ,, | Yes | 30 | 30 | ,, ,, |
| 950r ,, | Yes | 30 | 80 | Intestinal damage and haematopoietic failure. |
| 850r ,, | Yes | 30 | 95 | — |

*Tumour.*—The tumour was maintained in an ascitic form, being passaged about every second week. For experimental purposes the ascitic fluid was taken ten days after implantation and after appropriate dilution with isotonic saline was injected intravenously into recipient mice, each receiving approximately $3 \times 10^6$ nucleated cells. In a few animals the tumour was maintained as a solid subcutaneous implant.

*Bone marrow.*—Where bone marrow was given after Degranol or irradiation it was removed from the femurs of CBA female donors, suspended in isotonic saline, and injected intravenously into the treated host mice. Four femur equivalents of bone marrow were injected into five mice. This corresponds to a dose per mouse of 10–12 $\times$ $10^6$ nucleated cells. Bone marrow was injected within 6 hours of irradiation and between 6 to 24 hours after treatment with Degranol. It was assumed on chemical grounds that no Degranol would be available for reaction with the donor cells 6 hours after its injection.

*Experiments.*—A few preliminary experiments were necessary to determine the correct dosage of both Degranol and irradiation, the results of which are summarised in Table I.

It can be seen that syngeneic bone marrow is effective therapy after 850 r X-rays and moderately effective after 80 mg./kg. Degranol. It seems that the Degranol had some toxic effects against which bone marrow was of no avail. On the grounds of previous experience with this and other tumours it was decided to treat mice 5 days after injection with tumour. It was known that after intravenous injection the NK/Ly is rarely obvious as a visible growth until at least 20 days later. Usually at this time lymph nodes, particularly in the region of the head and neck, become visibly swollen and this is followed later by enlargement of the thymus and eventually by growths in almost every part of the body. Most but not all mice are killed within 70 days of their implantation with the tumour. As will be seen the tumour is not invariably lethal. The results of the first experiments are illustrated in Fig. 1.

The salient feature of this experiment was that the treated animals died before the controls. Post mortem examinations were carried out and in every case the bone marrow appeared to be of normal cellularity. Nearly all the treated animals showed signs of the tumour at death and many of them died of asphyxiation due to enlargement of the thymus. One remarkable finding was that the lymph nodes in some if not all of the treated animals were atrophic even 20 or more days after treatment. Atrophy at this time is not normally observed in syngeneic bone marrow chimaeras. From this first experiment it was obvious that neither radiation nor Degranol was sufficient to kill the tumour at the doses used. It was less obvious but possible that the tumour and its host were in immunological conflict. In the light of these results two further experiments were planned.

1. It was intended to give either irradiation or Degranol followed by syngeneic bone marrow and, 9 days later, at a time when recolonization of the bone marrow is usually complete, Degranol to the irradiated mice and irradiation to the Degranol-treated mice, again followed by bone marrow. In this way it was hoped to kill more tumour cells than by one treatment. The treatments were to be reversed because the animals were not likely to tolerate two doses of Degranol. The first treatments were given to tumour-bearing mice but 9 days later their general condition was poor and it was thought unlikely that they would survive a second treatment. Blood counts revealed a severe anaemia and it was decided to give the animals an injection of syngeneic spleen cells. It was expected that this measure might remedy the apparent haematopoietic deficiency and, as the spleen contains a high proportion of lymphoid cells, might also check the growth of the tumour. The animals died at the same time as had animals given a single injection of bone marrow (Fig. 1), and the post mortem findings were similar.

2. If the host animals had had any immunological reaction against the tumours then the use of putatively immune animals as donors might have been helpful. A group of prospective donors were given a large subcutaneous implant of a solid form of the NK/Ly. One week later their bone marrow was injected intravenously to tumour bearing mice after either irradiation or Degranol. The spleen and axillary and inguinal lymph nodes of the bone marrow donors were removed, teased out in saline, and injected intraperitoneally into the bone marrow recipients, each of which received a total of *circa* 100 $\times$ $10^6$ cells. It was hoped

FIG. 1.—The survival of CBA female mice after :—

(*a*) NK/Ly ascites on day 0.
(*b*) NK/Ly ascites on day −5, 80 mg./kg. Degranol, i.v., on day 0.
(*c*) NK/Ly ascites on day −5, 80 mg./kg. Degranol, i.v., followed by 12 $\times$ $10^6$ cells syngeneic bone marrow, i.v., on day 0.
(*d*) As (*c*) but with 850 r instead of Degranol.
(*e*) NK/Ly ascites on day −5, 80 mg./kg. Degranol, i.v., followed by 12 $\times$ $10^6$ cells, syngeneic bone marrow, i.v., on day 0, 50 $\times$ $10^6$ cells syngeneic spleen, i.v., on day 9.
(*f*) As (*e*) but with 850 r instead of Degranol.
At least 30 mice in each group.

that in these circumstances the hypothetical balance between growth of the tumour and the immune response of the host would be tipped in favour of the host. In fact all the treated animals died within 14 days (Fig. 2). They all showed haematopoietic aplasia. The tumour was not apparent but then it rarely is before 20 days after intravenous injection.

One further experiment was carried out. Two groups of CBA mice were injected intravenously with 3 $\times$ $10^6$ cells of NK/Ly ascites per mouse. The haematocrits and haemoglobin levels of one group were determined after various time intervals (Fig. 3). The other group, 5 days after injection of the tumours, were given 850 r followed shortly by 10 $\times$ $10^6$ cells of CBA bone marrow and their blood pictures were followed as before (Fig. 3). It was apparent that the presence of tumours induced a transient but considerable anaemia and that the

FIG. 2.—The survival of CBA mice after :—

(*a*) NK/Ly ascites on day 0.

(*b*) NK/Ly ascites on day −5, 80 mg./kg. Degranol, i.p., followed by $6 \times 10^6$ cells of syngeneic bone marrow, i.v., $+100 \times 10^6$ cells syngeneic spleen and lymph nodes, i.p., all from mice bearing a 7 day old subcutaneous implant of the NK lymphoma.

(*c*) As (*b*) but with 850 r instead of Degranol.

At least 30 mice in each group.

FIG. 3.—The haematocrits (PCV) and haemoglobin (Hb) levels of CBA female mice after intravenous injection of $3 \times 10^6$ cells of NK/Ly ascites.

- - -●- - - PCV }

- - -○- - - Hb } Tumour alone.

——●—— PCV }
——○—— Hb } Tumour on day 0, 850 r, total body followed by $10 \times 10^6$ cells syngeneic bone marrow on day 5.

effect of irradiation plus bone marrow injection was to intensify and prolong the anaemia. High mortality in the second group of mice, attributable to uncontrolled haemorrhage, limited the number bled to give the results indicated in the later part of the graph. Otherwise each point represents a mean from at least four mice, no mouse being bled more than once every 8 days.

### DISCUSSION

One interpretation of the experiments recorded depends on an investigation of the anaemia observed in tumour-bearing mice, with or without irradiation, in the last experiments. Such a study has been made (Davies, Cross and Lapis, 1962) revealing that a virus or virus-like entity associated with the tumour was the cause of the anaemia. It was shown that all cellular components of the blood were to some extent affected by the supposed virus and that the control of viral activity was apparently dependent on the immunological responsiveness of the infected host. Much remains to be clarified about the exact relationship between the tumour and the virus but sufficient is known to put forward a tentative explanation of the experimental results here obtained.

The first point is that as the virus tends to suppress, albeit temporarily, the lymphocyte count of tumour-bearing mice this in itself may facilitate the growth of the tumour and might perhaps explain the lack of strain specificity of the tumour. Irradiation, though destroying some tumour cells, would further deplete the lymphocyte count and the net effect might be accelerated growth of the tumour. The injection of bone marrow cells could not in the time available sufficiently increase the amount of lymphoid tissue available to modify this effect (see Koller, Davies and Doak, 1962, for a detailed consideration of the immunological consequences of injecting bone marrow after irradiation). The recovery of haemoglobin and haematocrit values by the 23rd day in irradiated tumour-bearing mice (Fig. 3) does, however, indicate that some degree of immunological control over viral activity is finally achieved. The deaths of irradiated tumour-bearing mice which had been given bone marrow and massive doses of spleen and lymph node cells from " immune " tumour bearing mice were probably due to the large numbers of virus particles likely to have been present in the donor inoculum.

Though it is not yet known to what extent viral multiplication takes place in the tumour cells it is possible that the sensitivity of the tumour to various chemotherapeutic agents (Németh and Kellner, 1961) is to some extent associated with viral destruction of the tumour ; the chemotherapeutic agents then serving slightly to depress the immune response and permit increased viral activity.* It would be necessary to assume in this case that any immunological activity of the host against its tumour is not so depressed as to accelerate growth of residual tumour cells as apparently has happened in the present series of experiments. It is also possible that damage to infected tumour cells by the chemotherapeutic agent might itself encourage viral growth (Lapis and Mercer, 1962) ; a quasi-induction phenomenon. Such speculation could well form the basis of further studies.

* It should, however, be emphasised that no evidence of viral activity has so far been discovered in those mice which have been used for the screening of chemotherapeutic agents against the NK/Ly ascites.

SUMMARY

An account is presented of unsuccessful attempts to cure the NK/Ly ascites tumour in mice by doses of Degranol or irradiation which, had they not been followed by syngeneic marrow therapy, would have proved lethal as a consequence of haematopoietic failure. Reasons for the lack of success are discussed and it is concluded firstly that even the high doses of anti-tumour agents given were insufficient to eradicate the tumour and, secondly, that the treated animals died before the controls because a virus or virus-like entity associated with the tumour seriously impeded haematopoietic recovery.

The authors are indebted to Professor P. C. Koller of the Chester Beatty Research Institute for his guidance throughout this work.

The senior author was supported financially by a Gordon Jacob Fellowship of the Royal Marsden Hospital for which he is very grateful and in other respects the work was supported by grants to the Chester Beatty Research Institute (Institute of Cancer Research : Royal Cancer Hospital) from the Medical Research Council, the British Empire Cancer Campaign, the Anna Fuller Fund, and the National Cancer Institute of the National Institutes of Health, U.S. Public Health Service.

### REFERENCES

Barnes, D. W. H., Corp, M. J., Loutit, J. F. and Neal, F. E.—(1956) *Brit. med. J.*, ii, 626.

*Idem* and Loutit, J. F.—(1957) *Brit. J. Haematol.*, **3**, 241.

Davies, A. J. S., Cross, A. M. and Lapis, K.—(1962) *Brit. J. Cancer*, **16**, 770.

Ford, C. E., Hamerton, J. L., Barnes, D. W. H. and Loutit, J. F.—(1956) *Nature, Lond.*, **177**, 452.

Hewitt, H. B. and Wilson, C. W.—(1959) *Brit. J. Cancer*, **13**, 69.

Koller, P. C., Davies, A. J. S. and Doak, S. M. A.—(1962) ' Advances in Cancer Research ', New York (Academic Press), p. 181.

Lapis, K. and Mercer, E. H.—(1962) *Cancer Res.*, in press.

Lorenz, E., Congdon, C. C. and Uphoff, D.—(1952) *Radiology*, **58**, 863.

Németh, L. and Kellner, B.—(1960) *Naturwissenschaften*, **47**, 544.—(1961) *Neoplasma*, **8**, 337.

Thomas, E. D., Lochte, H. L. and Ferrebee, J. W.—(1959) *Blood*, **14**, 1.

Uphoff, D. E.—(1957) *J. nat. Cancer Inst.*, **20**, 123.

*Idem* and Law, L. W.—(1958) *Ibid.*, **20**, 617.